# Investigating the association of traditional and non-traditional tobacco product use with subclinical and clinical cardiovascular disease: The Cross-Cohort Collaboration-Tobacco working group rationale, design, and methodology

**DOI:** 10.18332/tid/166517

**Published:** 2023-07-07

**Authors:** Erfan Tasdighi, Kunal K. Jha, Zeina A. Dardari, Ngozi Osuji, Tanuja Rajan, Ellen Boakye, Michael E. Hall, Carlos J. Rodriguez, Andrew C. Stokes, Omar El Shahawy, Emelia J. Benjamin, Aruni Bhatnagar, Andrew P. DeFilippis, Michael J. Blaha

**Affiliations:** 1 Johns Hopkins Ciccarone Center for Prevention of Cardiovascular Disease, Baltimore, United States; 2 American Heart Association Tobacco Regulation and Addiction Center, Dallas, United States; 3 Department of Medicine, University of Mississippi Medical Center, Jackson, United States; 4 Division of Cardiology, Department of Medicine, Albert Einstein College of Medicine, New York, United States; 5 Department of Global Health, School of Public Health, Boston University, Boston, United States; 6 Department of Population Health, New York University Grossman School of Medicine, New York, United States; 7 Cardiovascular Medicine, Boston Medical Center, Boston University School of Medicine, Boston, United States; 8 Department of Epidemiology, School of Public Health, Boston University, Boston, United States; 9 School of Medicine, University of Louisville, Louisville, United States; 10 Department of Medicine, Vanderbilt University Medical Center, Nashville, United States

**Keywords:** cross-cohort collaboration, tobacco smoking, noncigarette tobacco, e-cigarette, cardiovascular disease

## Abstract

While the impact of combustible cigarette smoking on cardiovascular disease (CVD) is well-established, the longitudinal association of non-traditional tobacco products with subclinical and clinical CVD has not been fully explored due to: 1) limited data availability; and 2) the lack of well-phenotyped prospective cohorts. Therefore, there is the need for sufficiently powered well-phenotyped datasets to fully elucidate the CVD risks associated with non-cigarette tobacco products. The Cross-Cohort Collaboration (CCC)-Tobacco is a harmonized dataset of 23 prospective cohort studies predominantly in the US. *A priori* defined variables collected from each cohort included baseline characteristics, details of traditional and non-traditional tobacco product use, inflammatory markers, and outcomes including subclinical and clinical CVD. The definitions of the variables in each cohort were systematically evaluated by a team of two physician-scientists and a biostatistician. Herein, we describe the method of data acquisition and harmonization and the baseline sociodemographic and risk profile of participants in the combined CCC-Tobacco dataset. The total number of participants in the pooled cohort is 322782 (mean age: 59.7 ± 11.8 years) of which 76% are women. White individuals make up the majority (73.1%), although there is good representation of other race and ethnicity groups including African American (15.6%) and Hispanic/Latino individuals (6.4%). The prevalence of participants who never smoked, formerly smoked, and currently smoke combustible cigarettes is 50%, 36%, and 14%, respectively. The prevalence of current and former cigar, pipe, and smokeless tobacco is 7.3%, 6.4%, and 8.6%, respectively. E-cigarette use was measured only in follow-up visits of select studies, totaling 1704 former and current users. CCC-Tobacco is a large, pooled cohort dataset that is uniquely designed with increased power to expand knowledge regarding the association of traditional and non-traditional tobacco use with subclinical and clinical CVD, with extension to understudied groups including women and individuals from underrepresented racial-ethnic groups.

## INTRODUCTION

Cardiovascular disease (CVD) is the leading cause of death in the US and globally, producing significant health and economic burden^1,2^. Combustible cigarette smoking is a well-established independent risk factor for CVD^2-5^. Leveraging such evidence, coupled with robust regulatory policies and enforcement, have resulted in a steady decline in combustible cigarette use across different population subgroups in the US^6,7^.

Despite the decrease in the rates of smoking, the popularity of non-cigarette tobacco products has increased in the past few decades^8-10^. Between 2000 and 2015, smokeless tobacco use among US adults increased by 23%8. In 2020, 2.3% of US adults reported past 30-day smokeless tobacco use, while 1.6% of youth reported smokeless tobacco use in 2022^6,11^. Despite a reduction in cigar use in some subgroups, use has increased 68% among adult women^12^. Additionally, cigar use has decreased among older adults but increased from 12.0% in 2002 to 12.7% in 2008 among those aged 18–25 years^13^. Lastly, the use of e-cigarettes has become increasingly popular, with approximately 5.1% of US adults reporting past 30-day use of e-cigarettes in 2020^14^. Despite the significant increase in the use of non-traditional tobacco products, important knowledge gaps on their health effects remain, and several studies have reported mixed results on the association of these non-traditional tobacco products and CVD risk^14,15^.

The use of longitudinal data such as the Population Assessment of Tobacco and Health (PATH) has been instrumental in studying the potential health effects of newer tobacco products such as e-cigarettes^15,16^. The PATH study is, however, limited by self-reported, non-adjudicated outcomes that could result in misclassification, short follow-up period, and the low prevalence of non-cigarette tobacco product use^17^. Given the relatively low prevalence of non-traditional tobacco products in individual prospective cohort studies, the synthesis of various datasets can lead to the construction of high-powered and phenotypically diverse databases of unparalleled size. Therefore, prioritizing data synthesis from multiple existing cohorts can offset the financial, technical, and time constraints related to developing new well-powered studies, which supported the rise of large consortia like the Cross-Cohort Collaboration (CCC)^18^.

The CCC was instituted to develop the infrastructure, policies, and design procedures for harmonization and eventual data sharing for the purpose of studying chronic disease epidemiology. The objective of the tobacco working group arm of the CCC is to provide additional insight into the cardiovascular health implications of non-cigarette tobacco product use with an emphasis on subclinical and clinical CVD.

The 2016 Tobacco Deeming rule extended the regulatory authority of the US Food and Drug Administration (FDA) to include the manufacturing, marketing, and distribution of non-cigarette tobacco products, including e-cigarettes, pipe tobacco, cigars, hookah/waterpipe tobacco, and e-liquids^19^. The CCC-Tobacco, which is partly supported by the Tobacco Centers of Regulatory Science (TCORS) program, funded by the Center for Tobacco Products of the FDA, seeks to inform the regulatory efforts of the agency directed towards non-traditional tobacco products. The CCC-Tobacco received ethical approval from the Johns Hopkins institutional review board. This article describes the design and methodology for creating and harmonizing the CCC-Tobacco dataset and presents the distribution of baseline sociodemographic characteristics and tobacco exposure in CCC-Tobacco.

## METHODS

### Cohorts that comprise the CCC-Tobacco

**Figure 1:**
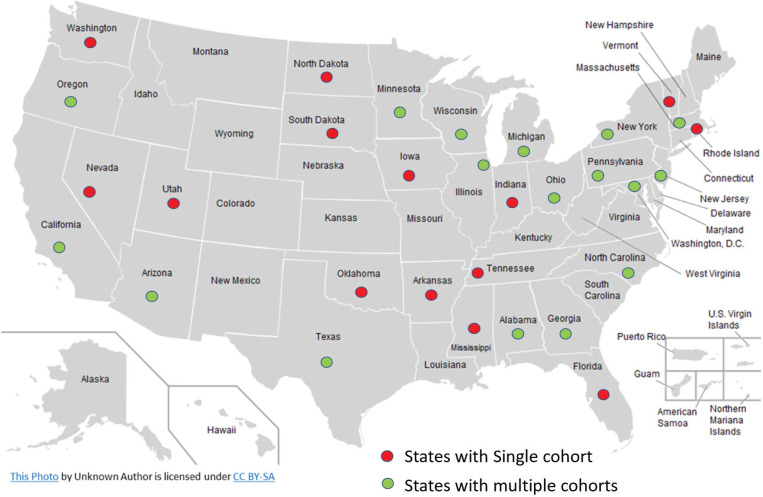
Geographical distribution of participating cohorts’ investigations sites Cross-Cohort Collaboration-Tobacco dataset

Twenty-three prospective observational cohort studies in the US and Brazil with baseline and follow-up data on tobacco use have currently provided de-identified individual-level data to the CCC-Tobacco. These include nine landmark cohorts which were originally designed to study CVD epidemiology (i.e. traditional cardiovascular cohorts): Atherosclerosis Risk in Communities (ARIC) Study, Coronary Artery Risk Development in Young Adults (CARDIA) Study, Cardiovascular Health Study (CHS), Dallas Heart Study (DHS), Framingham Heart Study (FHS), Hispanic Community Health Study/Study of Latinos (HCHS/SOL), Jackson Heart Study (JHS), Multi-Ethnic Study of Atherosclerosis (MESA), Multiple Risk Factor Intervention Trial (MRFIT), the Reasons for Geographic and Racial Differences in Stroke Study (REGARDS), and the Strong Heart Study (SHS). Other cohorts included in the CCC-Tobacco are (non-cardiovascular specific cohorts): Baltimore Longitudinal Study of Aging (BLSA); Chronic Renal Insufficiency Cohort (CRIC); the Brazilian Longitudinal Study of Adult Health (ELSA-Brasil); Genetics of Lipid Lowering Drugs and Diet Network (GOLDN); the Health, Aging and Body Composition Study (Health ABC); the Osteoporotic Fractures in Men Study (MrOS); Rancho Bernardo Study (RBS) of Healthy Aging; Study of Osteoporotic Fractures (SOF); the Study of Women’s Health Across the Nation (SWAN); and Women’s Health Initiative (WHI). Characteristics of participating cohorts and their geographical distribution are presented in Table 1 and Figure 1, respectively. For additional details, including study-specific rationale, design, funding, and protocols, and appropriate links to background reading, are given in Supplementary file Table 1. Additionally, the contribution of each participating cohort to the whole CCC-Tobacco dataset is given in Supplementary file Figure 1.

**Table 1:** Characteristics of the twenty-three participating cohorts of the Cross-Cohort Collaboration-Tobacco dataset

*Participating cohorts (website link)*	*Cohort population and description*	*Enrollment years*
**Traditional cardiovascular cohorts**		
Atherosclerosis Risk in Communities Study (ARIC)	15792 in 4 US communities aged 45–64 years	1987
Coronary Artery Risk Development in Young Adults (CARDIA)	5115 at 4 US field centers aged 18–30 years	1985–86
Cardiovascular Health Study (CHS)	5888 adults aged ≥65 years in 4 US communities	1989–1999
Dallas Heart Study (DHS)	6101 from multi-ethnic cohort of Dallas County	2000
Framingham Heart Study (FHSL)	5209 adult population of Framingham Massachusetts aged 30–62 years (original cohort)	1948
	Offspring cohort: 5124 adult children of the original cohort and their spouses aged 30–74 years	1971
	FHS 3^rd^ Gen: 4095 men and women aged >19 years with ≥one parent in the offspring study	2002
Hispanic Community Health Study/Study of Latinos (HCHS-SOL)	16000 persons of Hispanic/Latino origin from 4 field US centers aged 18–74 years	2006
Jackson Heart Study (JHS)	5306 community-based African Americans from 3 counties in Jackson MS aged 35–84 years	2000–2004
Multi-Ethnic Study of Atherosclerosis (MESA)	More than 6000 multi-ethnic men and women from 6 communities in the US aged 45–84 years	2000–2002
The Multiple Risk Factor Intervention Trial (MRFIT)	12866 men aged 35–57 years enrolled in coronary heart disease intervention trial	1972
Reasons for Geographic and Racial Differences in Stroke (REGARDS)	30239 employed men and women aged ≥45 years	2003
Strong Heart Study (SHS)	4500 American Indian tribal members aged 35–74 years	1988
**Non-cardiovascular specific cohorts**		
Baltimore Longitudinal Study of Aging (BLSA)	>3000 men and women aged >20 years	1958
Chronic Renal Insufficiency Cohort Study (CRIC)	3939 with chronic kidney disease (1560 older adults during third phase)	2001–2013 (I & II) 2013–2015 (III)
Brazilian Longitudinal Study of Adult Health (ELSA-Brasil)	15000 active and retired civil servants from teaching and research institutions aged 35–74 years	2008
Genetics of Lipid Lowering Drugs and Diet Network (GOLDN)	1200 white family members from 2 genetically homogeneous US centers aged >18 years	2004–2006
Health Aging and Body Composition Study (Health ABC)	3075 community-dwelling in Memphis TN or Pittsburgh PA and aged 70–79 years	1997
The Osteoporotic Fractures in Men Study (MrOS)	6000 senior men ≥65 years from 6 US communities	2000
Rancho Bernardo Study (RBS) of Healthy Aging	6339 Community based cohort of all residents of Rancho Bernardo	1972–1974
The Study of Osteoporotic Fractures (SOF)	10366 older women aged ≥65 years	1986
Study of Women’s Health Across the Nation (SWAN)	3302 women in longitudinal study of women’s health in 7 US research centers	1996–1997
Women’s Health Initiative (WHI)	161808 postmenopausal women aged 50–79 years	1993
**Total population (N)**	**322782**	**1948–2015**

Most of the studies began recruiting participants between 1948 and 2008. Four of the cardiovascular studies (ARIC, CARDIA, DHS, MESA) specifically recruited participants from different racial groups, and three were designed to primarily study specific racial or ethnic groups (Hispanic/Latino participants in HCHS/SOL, Black participants in JHS, and Indigenous participants in SHS). The WHI is one of the largest women’s health projects ever launched in the US, having enrolled more than 161000 women aged 50–79 years at 40 clinical centers. The main areas of research were CVD, cancer, and osteoporotic fractures in postmenopausal women.

All the cohorts have extensive data on participants’ baseline sociodemographic characteristics, and gather data on participant tobacco use behaviors, although this varies in scope and detail. Many cohorts that comprise the CCC-Tobacco have collected detailed information on participants’ health and behavior for as long as fifty years of follow-up. Twenty-one cohorts (except ELSA-Brasil and SOF) ascertain CVD including myocardial infarction, stroke, atrial fibrillation, and heart failure, and several cohorts report measures of subclinical cardiovascular injury including measures of inflammation, coronary artery calcium (CAC), carotid plaque, carotid intima-media thickness (cIMT), pulse-wave velocity, and ankle-brachial index.

### Participants

Cohort participants previously provided informed consent for in-person, telephone, and/or email contact and for the abstraction of medical records. The institutional review board at each research center approved the study protocol for each cohort. The twenty-three cohorts in the consortium provided data from approximately 322000 participants. All forty-eight continental US states are represented among CCC-Tobacco participants, including rural, suburban, and urban communities (Figure 1). In all, the cohorts included in the CCC-Tobacco have been or are being conducted across approximately forty field/clinical centers. One cohort with extensive geographical reach, the REGARDS, operates via telephone and in-home exams only.

### CCC-Tobacco variable domains

We requested and obtained individual-level de-identified data from all participating studies based on the following variable list. Baseline characteristics included sociodemographic variables such as age, sex, race/ethnicity, study site, education status, and income level. Past medical history, family history, and anthropometric variables including body mass index (BMI) were also requested. Measured cardiometabolic parameters including systolic blood pressure (SBP), diastolic blood pressure (DBP), total cholesterol (TC), high-density lipoprotein (HDL) cholesterol, low density lipoprotein (LDL) cholesterol, lipoprotein a [Lp(a)], and triglycerides data were requested. Data on the use of lipid-lowering therapy, anti-hypertensive therapy, anti-hyperglycemic medications, and anti-platelet medications were also collected.

Furthermore, self-reported health behaviors such as physical activity, diet, and the use of traditional and non-traditional tobacco products were requested from all the cohorts. Comorbidities were defined as follows. Obesity was defined as BMI ≥30 kg/m^2^. Hypertension was defined as SBP ≥140 mmHg, DBP ≥90 mmHg, or use of hypertensive medications. Diabetes was defined as a fasting blood glucose level ≥126 mg/dL, previous diagnosis of diabetes (treated or untreated), or use of antidiabetic medications. Dyslipidemia was defined as if one the following were present: 1) TC >240 mg/dL; 2) Triglycerides >200 mg/dL; 3) HDL-C <50 mg/dL (female) or <40 mg/dL (male); 4) LDL-C >160 mg/dL; or 5) the use of lipid lowering therapies. Hyperlipidemia was defined as either: 1) TC >240 mg/dL; 2) Triglycerides >200 mg/dL; or 3) LDL-C >160 mg/dL.

Participating studies provided baseline and longitudinal data over multiple study visits on the use of cigarettes, cigars, pipes, smokeless tobacco, and e-cigarettes, as well as secondhand smoke exposure. Data on the intensity and duration of exposure including tobacco-product use-years and usage per day were also collected when available. Additionally, data on the patterns and changes in tobacco use over time such as poly-product use, product switching, and quitting were collected.

Biomarkers of subclinical cardiovascular injury based on three domains – subclinical inflammation, thrombosis, and atherosclerosis – were collected. Inflammatory biomarkers included high-sensitivity C-reactive protein (hsCRP) and interleukin-6. Thrombosis biomarkers included fibrinogen and D-dimer. Measures of atherosclerosis included CAC, carotid plaque, cIMT test readings, pulse-wave velocity, and ankle-brachial index. The most recent data on cardiovascular outcomes were requested from each participating study. The outcomes included cardiovascular events (myocardial infarction, stroke, atrial fibrillation, heart failure) and mortality (coronary, cardiovascular, and all-cause). Furthermore, harmonized time-to-event variables will be constructed for the purpose of future survival analysis.

### Data acquisition and transfer

**Figure 2:**
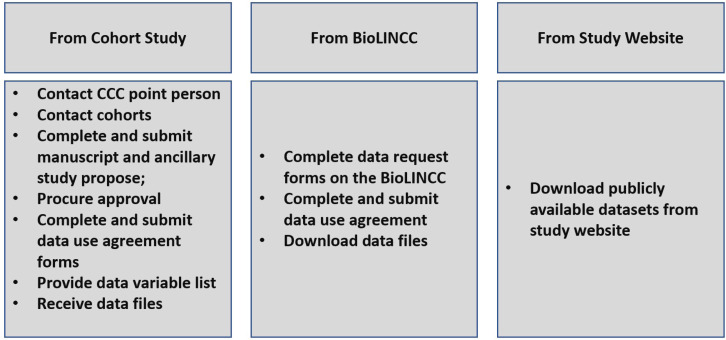
Steps in the data acquisition process Cross-Cohort Collaboration-Tobacco dataset

The data acquisition process consisted of establishing contact with the designated contact for each cohort, who then advised on the preferred mode of data transfer for the cohort. For most of the studies, the process entailed reaching out to the designated contact and subsequently submitting a study proposal which was then peer reviewed and ultimately approved by the cohort administrators or returned with request for changes. Upon approval of the proposal, data use agreements were completed and signed. Subsequently, data variable lists were sent to each study contact person. For studies like the FHS, data were obtained from the Biologic Specimen and Data Repository Information Coordinating Center (BioLINCC). Finally, several cohorts’ datasets were downloaded directly from the study website including the RBS and HEALTHABC. Upon transfer, datasets were stored in a secure encrypted cloud space (SafeDesktop) at the Johns Hopkins University School of Medicine. The process of data acquisition is summarized in Figure 2.

### Data harmonization

**Figure 3:**
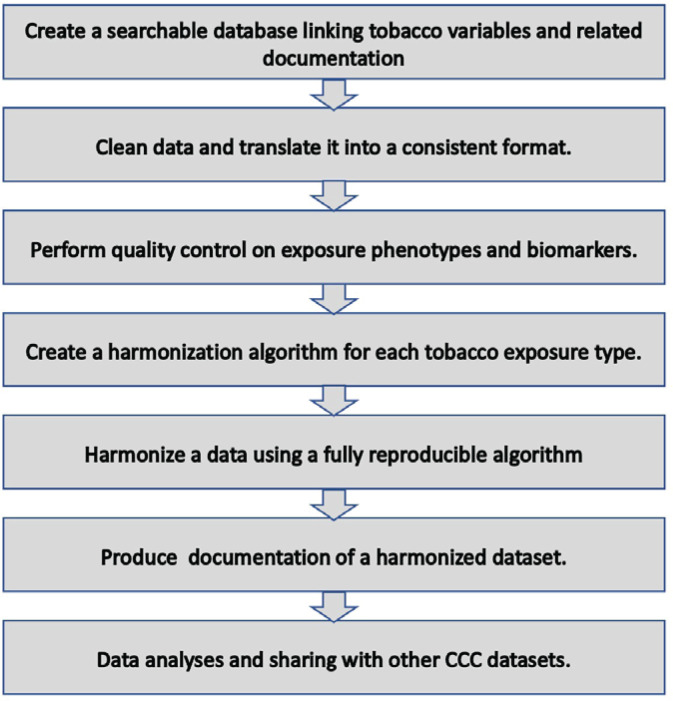
Data harmonization plan Cross-Cohort Collaboration-Tobacco dataset

Data management and harmonization was conducted centrally at the Johns Hopkins University School of Medicine. Upon the receipt of datasets, data were checked for missing variables and any other inconsistencies following which the data providers for the respective study were queried. The decision to harmonize a variable was made if the given variable had been provided by more than one study. Our harmonization techniques were informed by Maelstrom, a McGill University-based group at the forefront of innovative methodological approaches to harmonization. Maelstrom published the first harmonization guidelines and pioneered tools to facilitate documentation, harmonization, and integration^20^. Additionally, we iteratively learned from the data harmonization methods used for the Trans-Omics for Precision Medicine (TOPMed) project^21^, an NHLBI-funded effort to couple whole-genome sequencing (WGS) and other Omics data (e.g. DNA methylation signature, RNA expression, and metabolite profiles) with molecular, behavioral, imaging, environmental, and clinical data. We also leveraged some of the techniques applied in the Lifetime Risk Pooling Project (LRPP)^22^, which combines 20 US community cohorts in a life course study, and the International Collaboration for a Life Course Approach to Women’s Reproductive Health and Chronic Disease Events (InterLACE)^23^, which harmonized 20 cohorts across ten countries. Figure 3 provides a simplified schematic framework of the current data harmonization process.

### Statistical analysis

The association between smoking and CVD will be analyzed using survival analysis (COX proportional hazard model). In terms of studying the association of tobacco use transitions and CVD outcomes, our team has pioneered an approach that divides each participant’s experience into ‘person-trials’ reflecting tobacco use exposures accruing between each study visit. We have used this approach in one of our peer-reviewed publications^16^. This technique uses a variation of latent class mixed models (LCMM).

### Preliminary results

The CCC-Tobacco includes approximately 322000 participants from 23 predominantly NHLBI-funded prospective cohort studies. The baseline characteristics of the study participants are presented in Table 2. The mean age ± SD at baseline examination for the combined cohort is 59.7 ± 11.8 years and about three-quarters of the participants are women (76%). CARDIA and FHS Offspring studies have relatively younger participants with mean age of 29.9 ± 3.6 and 36.8 ± 9.9 years, respectively, and the oldest is CHS with a mean age of approximately 72 years at baseline. The overall population is predominantly White (73.1%); the rest of the cohort is 15.6% African American, 6.4% Hispanic/Latino, 1.8% Asian, and 2.8% are American Indian or Alaskan Native participants. Almost all the participants enrolled in the FHS are White, and MESA is a racially and ethnically diverse group with 38.5% White, 27.8 % African American, 12% Chinese American, and 22% Hispanic/Latino participants. About one-fifth of the entire cohort (22.8%) completed high school education while 64.9% have at least some college education, with considerable variation across the cohorts.

**Table 2:** Baseline characteristics across the thirteen traditional cardiovascular cohorts in the Cross-Cohort Collaboration-Tobacco dataset (Part 1)

*Characteristics*	*ARIC*	*CARDIA*	*CHS*	*DHS*	*FHS original*	*FHS off*	*FHS gen*	*HCHS-SOL*	*JHS*	*MESA*	*MRIFT*	*REGARDS*	*SHS*
**Sample size**	15776 (4.89)	4341 (1.34)	5882 (1.61)	2415 (0.75)	3885 (1.20)	4838 (1.50)	4061 (1.26)	16322 (5.06)	5218 (1.62)	6792 (2.10)	12866 (3.99)	30067 (9.31)	3389 (1.05)
**Age** (years)	54.2 ± 5.76	29.9 ± 3.6	72.7 ± 5.6	43.4 ± 10.5	55.5 ± 8.4	36.8 ± 9.89	40.1 ± 8.8	45.8 ± 13.9	54.8 ± 12.8	62.1 ± 10.2	46.2 ± 5.9	64.8 ± 9.42	56.5 ± 8.16
**Female**	8710 (55.1)	2393 (54.9)	3390 (57.6)	1388 (57.4)	2296 (51.6)	2509 (51.6)	2168 (53.3)	9790 (59.9)	3367 (63.4)	3601 (52.5)	0 (00.0)	16567 (55.1)	1982 (58.4)
**Race/ethnicity**													
White	11478 (72.7)	2224 (51.3)	4922 (84.0)	777 (32.1)	3885 (100)	4838 (100)	4061 (100)	0	0	2615 (38.5)	11559 (89.8)	17614 (58.5)	0
African American	4258 (27.0)	2117 (48.7)	921 (15.7)	1248 (51.6)	0	0	0	0	5128 (100)	1879 (27.8)	931 (7.2)	12453 (41.4)	0
Asian	34 (0.2)	0	4 (0.07)	0	0	0	0	0	0	802 (11.8)	0	0	0
Hispanic/Latino	0	0	0	344 (14.2)	0	0	0	16322 (100)	0	1496 (21.9)	0	0	0
American Indian or Alaskan	14 (0.1)	0	15 (0.3)	0	0	0	0	0	0	0	0	0	3389 (100)
Other	0	0	0	46 (1.9)	0	0	0	0	0	0	376 (2.9)	0	0
**Education**													
High school	3767 (23.9)	197 (4.5)	1730 (29.5)	384 (15.9)	1620 (41.0)	304 (8.9)	22 (0.65)	6189 (38.0)	973 (18.4)	1225 (18.0)	2083 (16.2)	3772 (12.5)	1438 (42.4)
High school completed	6412 (40.7)	2160 (49.8)	1583 (27.0)	717 (29.7)	1204 (30.5)	1719 (50.3)	465 (13.7)	4169 (25.6)	1065 (20.1)	1236 (18.2)	2685 (20.9)	7775 (25.8)	955 (28.2)
College degree	5586 (35.4)	1983 (45.7)	2552 (43.5)	1313 (54.3)	1123 (28.4)	1396 (40.8)	2897 (85.6)	5927 (36.4)	3248 (61.4)	4330 (63.8)	8035 (62.7)	18496 (61.5)	993 (29.3)
**Alcohol use**	8768 (55.8)	3606 (83.3)	2924 (49.9)	1707 (70.8)	2892 (71.4)	4166 (86.6)	3367 (82.9)	7733 (47.4)	2419 (45.8)	3749 (68.4)	11897(92.5)	10999 (37.3)	0 (0)
**Health status**													
BMI (kg/m^2^)	27.7 ± 5.37	26.1 ± 5.9	26.6 ± 4.7	9.5 ± 7.0	25.8 ± 4.1	25.2 ± 4.31	26.9 ± 5.6	29.7 ± 6.0	54.8 ± 12.8	28.3 ± 5.47	27.7 ± 3.4	29.3 ± 6.2	30.4 ± 6.0
Hypertension	5506 (34.9)	195 (4.5)	3886 (66.1)	810 (33.6)	1877 (48.4)	951 (19.7)	673 (16.6)	4446 (27.2)	3078 (59.1)	3285 (48.4)	11788 (91.6)	17782 (59.2)	1270 (37.6)
Systolic BP (mmHg)	121 ± 18	108 ± 11	137 ± 22	124 ± 17	138 ± 23	122 ± 16	117 ± 14	122 ± 18	127 ± 17	126 ± 21	148 ± 15	128 ± 17	126 ± 19
Diastolic BP (mmHg)	74 ± 11	69 ± 10	71 ± 12	79 ± 10	85 ± 11	79 ± 11	75 ± 10	73 ± 11	76 ± 9	72 ± 10	99 ± 7	76 ± 10	76 ± 10
BP medication	4004 (29.9)	70 (1.6)	2787 (47.5)	498 (20.6)	377 (9.3)	160 (3.3)	343 (8.5)	2645 (16.5)	2454 (52.4)	2536 (37.2)	2488 (19.4)	15490 (53.6)	376 (18.4)
Diabetes	1561 (9.9)	83 (1.92)	925 (16.0)	220 (9.2)	171 (4.2)	91 (1.9)	123 (3.0)	3235 (20.1)	1242 (23.7)	859 (12.6)	711 (5.6)	6378 (22.0)	1387 (41.2)
Dyslipidemia	8986 (57.8)	1379 (32.5)	2955 (50.7)	1086 (50.5)	2355 (60.1)	2221 (46.5)	1557 (38.3)	9740 (60.2)	2828 (57.6)	3787 (55.7)	10391 (80.8)	19105 (65.4)	2118 (63.5)
HPL	5771 (37.1)	411 (9.7)	1947 (33.4)	378 (17.6)	2346 (59.8)	1135 (23.8)	671 (16.5)	4492 (27.8)	1124 (23.3)	1559 (22.9)	8970 (69.7)	6802 (23.60	882 (26.5)
HTG	4269 (27.4)	330 (7.8)	1816 (31.2)	496 (23.1)	652 (38.8)	684 (14.3)	846 (20.8)	5070 (31.4)	776 (16.1)	1988 (29.3)	6903 (53.67)	8004 (27.8)	1112 (33.4)
HPL medication	448 (2.9)	11 (0.2)	132 (2.2)	151 (6.2)	46 (1.1)	27 (0.6)	273 (6.7)	1981 (12.4)	721 (13.7)	1100 (16.1)	159 (1.2)	9977 (33.5)	12 (0.59)
LDL-C (mg/dL)	137.6 ± 39.3	108.5 ± 32.0	129.8 ± 35.6	106.8 ± 34.9	-	128.6 ± 37.2	111.7 ± 31.4	122.6 ± 36.6	126.6 ± 36.6	117.2 ± 31.4	160.0 ± 36.0	113.9 ± 34.8	110.3 ± 31.9
Total cholesterol (mg/dL)	214.9 ± 42.0	178.1 ± 34.3	211.2 ± 39.2	181.0 ± 38.4	252.9 ± 48.3	200.2 ± 40.0	188.8 ± 35.5	199.2 ± 44.1	199.3 ± 40.1	194.1 ± 35.7	240.4 ± 36.8	192.0 ± 40.1	195.4 ± 39.6
HDL-C (mg/dL)	214.9 ± 42.0	53.3 ± 14.1	54.1 ± 15.7	50.4 ± 14.7	-	51.8 ± 16.2	54.3 ± 16.1	49.2 ± 13.0	51.8 ± 14.6	50.9 ± 14.8	42.0 ± 11.7	51.7 ± 16.1	46.1 ± 13.8
Triglycerides (mg/dL)	131.8 (79–157)	80.8 (46–94)	139.6 (86–161)	122.6 (67–145)	152 (98–178)	100 (56–120)	116 (65–138)	139.7 (80–166)	106.4 (65–126)	132 (78–161)	194 (113–228)	132 (81–158)	150 (82–172)

*Data are presented as frequency and percentage n (%), mean ± standard deviation, or mean (range). ARIC: Atherosclerosis Risk in Communities Study. CARDIA: Coronary Artery Risk Development in Young Adults Study. CHS: Cardiovascular Health Study. DHS: Dallas Heart Study. FHS: Framingham Heart Study. HCHS/SOL: Hispanic Community Health Study/Study of Latinos. JHS Jackson Heart Study. MESA: Multi-Ethnic Study of Atherosclerosis. MRFIT: Multiple Risk Factor Intervention Trial. REGARDS: the Reasons for Geographic and Racial Differences in Stroke Study. SHS: the Strong Heart Study. BMI: body mass index. BP: blood pressure. HPL: hyperlipidemia. HTG: hypertriglyceridemia. LDL-C: low density lipoprotein cholesterol. HDL-C: high density lipoprotein cholesterol.

**Table 2:** Baseline characteristics across the ten non-cardiovascular cohorts in the Cross-Cohort Collaboration-Tobacco dataset (Part 2)

	*BLSA*	*CRIC*	*ELSA*	*GOLDEN*	*Health ABC*	*MROS*	*RBS*	*SOF*	*SWAN*	*WHI*	*Total*
**Sample size**	1788 (0.55)	4917 (1.52)	15104 (4.68)	958 (0.30)	3070 (0.95)	5993 (1.86)	2475 (0.77)	9673 (3.00)	3270 (1.01)	159682 (49.47)	322782 (100)
**Age** (years)	65.7 ± 15.1	59.1 ± 10.7	52.0 ± 9.0	48.2 ± 16.4	73.6 ± 2.87	73.6 ± 5.87	70.1 ± 11.0	71.6 ± 5.22	45.8 ± 2.6	63.2 ± 7.2	59.7 ± 11.8
**Female**	924 (51.6)	2121 (43.1)	82185 (4.41)	506 (52.8)	1582 (51.5)	0 (0.00)	1382 (55.8)	9673 (100)	3270 (100.0)	159682 (100)	245405 (76.0)
**Race/ethnicity**											
White	1268 (74.2)	2010 (42.4)	-	942 (98.3)	1792 (58.3)	5384 (89.8)	2448 (100)	9640 (100)	1546 (47.2)	135962 (85.1)	224957 (73.1)
African American	405 (23.7)	2130 (44.9)	-	0	1278 (41.6)	244 (4.0)	0	0	914 (27.9)	14019 (8.8)	48015 (15.6)
Asian	30 (1.7)	0	-	2 (0.2)	0	192 (3.2)	0	0	529 (16.1)	4002 (2.5)	5595 (1.8)
Hispanic/Latino	0	601 (12.6)	-	10 (1.0)	0	98 (1.6)	0	0	281 (8.5)	525 (0.3)	19677 (6.4)
American Indian or Alaskan	5 (0.2)	0	-	0	0	68 (1.1)	0	0	0	5174 (3.2)	8665 (2.8)
Other	0	0	-	4 (0.4)	0	7 (0.1)	0	0	0	0	433 (0.1)
**Education**											
High school	17 (0.9)	1016 (20.6)	1921 (12.7)	-	774 (25.2)	393 (6.5)	149 (6.1)	2211 (22.9)	233 (7.1)	8463 (5.3)	38774 (12.1)
High school completed	148 (8.3)	916 (18.6)	5233 (34.6)	-	997 (32.5)	1036 (17.2)	613 (25.2)	3797 (39.3)	573 (17.6)	27291 (17.2)	72670 (22.8)
College degree	1612 (90.7)	2983 (60.6)	7950 (52.6)	-	1292 (42.1)	4564 (76.1)	1671 (68.6)	3636 (37.7)	2433 (75.1)	122751 (77.4)	206665 (64.9)
**Alcohol use**	1472 (82.5)	3090 (62.8)	7244 (47.9)	479 (50.0)	3067 (100)	3865 (64.6)	2188 (90.5)	6759 (69.9)	1335 (47.4)	-	93518 (59.5)
**Health status**											
BMI (kg/m^2^)	27.0 ± 4.9	32.2 ± 7.6	-	28.2 ± 5.7	27.3 ± 4.8	27.3 ± 3.8	24.8 ± 3.6	26.4 ± 4.4	28.2 ± 7.2	27.9 ± 5.9	28.1 ± 5.8
Hypertension	692 (39.5)	4275 (86.9)	5584 (37.0)	241 (25.2)	2112 (68.8)	4205 (70.9)	1471 (59.6)	6052 (62.59)	780 (23.9)	53578 (33.8)	134537 (41.9)
Systolic BP (mmHg)	118 ± 16	128 ± 22	124 ± 17	115 ± 17	136 ± 21	139 ± 19	139 ± 22	142 ± 19	118 ± 17	127 ± 18	128 ± 19
Diastolic BP (mmHg)	66 ± 9	71 ± 13	74 ± 10	68 ± 9	71 ± 12	-	76 ± 9	76 ± 9	75 ± 10	75 ± 9	76 ± 11
BP medication	632 (35.6)	4188 (97.0)	4411 (29.20	198 (20.6)	0 (0.0)	3053 (50.9)	776 (37.6)	2644 (30.3)	463 (14.2)	19459 (12.2)	70268 (22.4)
Diabetes	268 (15.0)	2464 (50.5)	3009 (19.9)	73 (7.6)	1012 (32.9)	881 (15.7)	257 (10.4)	681 (7.0)	126 (4.1)	9442 (5.9)	4130 (9.2)
Dyslipidemia	838 (50.7)	3920 (85.1)	8157 (54.0)	551 (57.6)	1385 (45.6)	3309 (58.5)	1072 (43.6)	551 (73.1)	1406 (43.3)	15831 (9.9)	23113 (52.2)
HPL	232 (14.8)	1086 (27.7)	5233 (34.7)	236 (24.7)	847 (27.9)	1475 (26.7)	909 (37.1)	450 (59.7)	573 (17.6)	-	13017 (29.5)
HTG	226 (14.4)	1479 (37.7)	4635 (30.7)	302 (31.60	918 (30.2)	2001 (36.2)	562 (22.9)	368 (48.8)	572 (18.6)	-	43951 (29.8)
HPL medication	528 (58.8)	3006 (61.6)	1978 (13.1)	144 (15.1)	437 (14.3)	1540 (25.7)	16 (0.8)	-	34 (1.1)	15831 (9.9)	35531 (12.5)
LDL-C (mg/dL)	109.6 ± 32.2	102.7 ± 35.5	-	121.1 ± 30.9	121.5 ± 34.6	114.1 ± 30.9	134.5 ± 36.8	152.0 ± 36.1	116.0 ± 30.9	-	124.3 ± 38.3
Total cholesterol (mg/dL)	189.8 ± 36.4	183.7 ± 45.5	-	189.7 ± 38.6	202.7 ± 38.5	193.2 ± 34.2	219.3 ± 40.4	239.1 ± 40.1	194.5 ± 34.8	-	202.2 ± 46.9
HDL-C (mg/dL)	59.6 ± 17.0	47.5 ± 15.4	-	46.9 ± 13.1	54.0 ± 17.0	48.9 ± 14.6	61.7 ± 18.7	53.1 ± 14.8	55.9 ± 14.5	-	50.6 ± 15.6
Triglycerides (mg/dL)	102 (66–122)	157 (89–186)	-	136 (73–171)	138 (88–163)	151 (91–179)	119 (69–145)	172 (106–207)	113 (67–131)	-	137 (79–163)

*Data are presented as frequency and percentage n (%), mean ± standard deviation, or mean (range). BLSA: Baltimore Longitudinal Study of Aging. CRIC: Chronic Renal Insufficiency Cohort. ELSA-Brasil: the Brazilian Longitudinal Study of Adult Health GOLDN: Genetics of Lipid Lowering Drugs and Diet Network. Health ABC: the Health Aging and Body Composition Study. MrOS: the Osteoporotic Fractures in Men Study. RBS: Rancho Bernardo Study of Healthy Aging. SOF: Study of Osteoporotic Fractures. SWAN: the Study of Women’s Health Across the Nation. WHI: Women's Health Initiative. BMI: body mass index. BP: blood pressure. HPL: hyperlipidemia. HTG: hypertriglyceridemia. LDL-C: low density lipoprotein cholesterol; HDL-C: high density lipoprotein cholesterol.

With respect to comorbidities, 29.5% reported having a history of hyperlipidemia and 9.2% diabetes mellitus. Mean SBP and DBP are 127 ± 19 and 75 ± 11 mmHg in the overall population. Self-reported use of blood pressure medication and lipid-lowering medication are 22.4% and 12.5%, respectively.

Smoking status of participants in each of the 23 cohorts is categorized into never, former, and current, for both combustible cigarettes and non-cigarette tobacco products including cigar, pipe, smokeless tobacco, and e-cigarette (Table 3). Overall, 46330 (14.3%) participants reported current use of combustible cigarettes and 117424 (36.4%) reported former use. The prevalence of current cigarette smoking is highest in MRFIT (63.6%) and lowest in MrOS (3.4%). Baseline characteristics of the participants based on their combustible cigarette smoking status are shown in Table 4. The mean age of individuals who reported current smoking is 53.4 ± 12.2 years compared to 59.8 ± 12.4 years for those who never smoked, or 62.1 ± 9.9 years who formerly smoked. The proportion of women is highest for individuals who never smoked (82.6%), followed by those who formerly smoked (75.0%), and those who currently smoke (55.9%). The prevalence of alcohol use is higher among participants who currently smoke compared to never smoked (74.2% vs 50.4%). Similarly, the prevalence of hypertension (44.1% vs 39.9%) and hyperlipidemia (39.4% vs 27.4%) is higher in participants who currently smoke compared to never smoked. The prevalence of diabetes is comparable, approximately 11% in both groups. Furthermore, more detail on smoking status based on race and ethnicity has been provided in Supplementary file Table 2.

**Table 3:** Distribution (%) of traditional and non-traditional tobacco products across all the cohorts in the Cross-Cohort Collaboration-Tobacco dataset

*Participating cohorts*	*Traditional cigarette status*			*Cigar*			*Pipe*			*Smokeless tobacco*			E-Cigarette*			*F/U visits*
	*Never*	*Former*	*Current*	*Never*	*Former*	*Current*	*Never*	*Former*	*Current*	*Never*	*Former*	*Current*	*Never*	*Former*	*Current*	
**Cardiovascular specific cohorts**																
ARIC	41.6	32.1	26.2	93.4	4.8	1.8	90.0	8.2	1.7	91.2	5.3	3.3				7
CARDIA	57.2	14.1	28.6	96.0	3.4	0.6	97.8	1.9	0.2	96.7	2.3	0.8	92.9	3.9	3.2	7
CHS	46.5	41.5	12.0													10
DHS	56.4	17.2	26.3	93.8	2.6	3.5	97.1	2.4	0.3	96.3	2.1	1.5				2
FHS original	44.0	9.8	46.1													8
FHS offspring	34.9	19.8	45.2	94.9	0.7	4.4	95.6	0.7	3.6							9
FHS 3^rd^ generation	57.1	27.3	15.5	98.4	0.5	1.1	99.2	0.7	0				94.6	3.9	1.5	2
HCHS-SOL	60.7	19.8	19.4										91.9	6.9	1.2	2
JHS	85.5	1.2	13.3	97.4	1.5	1.1	98.6	1.0	0.3	97.0	1.2	1.6				2
MESA	50.3	36.6	13.1	90.6	7.4	1.9	91.6	7.7	0.6	98.1	1.3	0.4	99.3	0.4	0.2	6
SHS	29.0	33.0	37.9													6
**Non cardiovascular specific cohorts**																
BLSA	60	5.6	34.1	90.9	7.2	1.9	88.1	11.5	0.3							7
CRIC	38.6	46.9	14.5	78.0	19.0	3.0				84.1	12.8	3.0				18
ELSA-Brasil	56.9	30.0	13.1													4
GOLDN	70.7	21.9	7.4													1
Health ABC	54.8	34.8	10.4													10
MRFIT	14.4	21.8	63.6													10
MrOS	37.5	59.0	3.4													10
RBS	45.2	32.3	22.4													12
REGARDS	45.2	40.1	14.6							88.4	9.4	2.1	98.0		2	2
SOF	60.4	29.6	10													7
SWAN	N/A	N/A	N/A													15
WHI	51.0	42.0	6.9													9
**Total estimated prevalence (N)**	**160000**	**117000**	**47000**	**43000**	**2400**	**1000**	**40000**	**2000**	**500**	**58000**	**4000**	**1400**	**37000**	**1000**	**600**	

ARIC: Atherosclerosis Risk in Communities Study. CARDIA: Coronary Artery Risk Development in Young Adults Study. CHS: Cardiovascular Health Study. DHS: Dallas Heart Study. FHS: Framingham Heart Study. HCHS/SOL: Hispanic Community Health Study/Study of Latinos. JHS Jackson Heart Study. MESA: Multi-Ethnic Study of Atherosclerosis. MRFIT: Multiple Risk Factor Intervention Trial. REGARDS: the Reasons for Geographic and Racial Differences in Stroke Study. SHS: the Strong Heart Study. BLSA: Baltimore Longitudinal Study of Aging. CRIC: Chronic Renal Insufficiency Cohort. ELSA-Brasil: the Brazilian Longitudinal Study of Adult Health GOLDN: Genetics of Lipid Lowering Drugs and Diet Network. Health ABC: the Health Aging and Body Composition Study. MrOS: the Osteoporotic Fractures in Men Study. RBS: Rancho Bernardo Study of Healthy Aging. SOF: Study of Osteoporotic Fractures. SWAN: the Study of Women’s Health Across the Nation. WHI: Women's Health Initiative.

* E-cigarette measures are only in follow-up visits of the respective cohorts.

**Table 4:** Baseline characteristics across combustible cigarette smoking status in the Cross-Cohort Collaboration-Tobacco dataset

*Characteristics*	*Never smoker*	*Former smoker*	*Current smoker*	*Total*
**Sample size**	159028 (49.3)	117424 (36.4)	46330 (14.3)	322782 (100)
**Age** (years)	59.8 ± 12.4	62.1 ± 9.9	53.4 ± 12.2	59.7 ± 11.8
**Female**	131395 (82.6)	88108 (75.0)	25902 (55.9)	245405 (100)
**Race/ethnicity**				
White	105907 (70.5)	89552 (79.4)	29498 (66.5)	29498 (66.6)
African American	24576 (16.4)	14548 (12.8)	8981 (20.3)	48015 (15.62)
Asian	4080 (2.7)	1274 (1.1)	241 (0.5)	5595 (1.80)
Hispanic/Latino	11743 (7.82)	4348 (3.85)	3586 (8.09)	19677 (6.40)
American Indian or Alaskan	3824 (2.5)	3026 (2.7)	1815 (4.1)	8665 (2.8)
Other	105 (0.07)	138 (0.1)	190 (0.4)	433 (0.1)
**Education**				
High school	18214 (11.6)	11781 (10.1)	8779 (19.4)	38774 (12.2)
High school completed	36059 (23.0)	23702 (20.0)	12909 (28.6)	72670 (22.8)
College degree	102490 (65.4)	80717 (69.50)	23458 (51.9)	206665 (64.97)
**Alcohol use**	37867 (50.40)	30788 (63.4)	24863 (74.2)	93518 (59.5)
**Health status**				
BMI (kg/m^2^)	28.2 ± 5.9	28.3 ± 5.8	27.1 ± 5.4	28.1 ± 5.8
Hypertension	63281 (39.9)	50857 (43.5)	20399 (44.1)	134537 (41.80)
Systolic BP (mmHg)	127.4 ± 18.9	128.3 ± 18.6	127.9 ± 20.2	127.8 ± 19.0
Diastolic BP (mmHg)	75 ± 10	76 ± 11	78 ± 13	76 ± 11
BP medication	34274 (22.1)	27026 (23.6)	8968 (20.2)	70268 (22.4)
Diabetes	16734 (10.6)	134 (11.5)	4975 (10.9)	35159 (10.9)
Dyslipidemia	46987 (59.9)	35906 (66.7)	22472 (64.9)	105365 (63.2)
HPL	19247 (27.4)	15014 (32.6)	13147 (39.4)	47402 (31.7)
HTG	17590 (25.4)	15114 (33.0)	11247 (34.9)	43951 (29.9)
HPL medication	17823 (11.7)	16679 (14.67)	4029 (9.1)	38531 (12.4)
LDL-C (mg/dL)	121.8 ± 36.7	123.1 ± 38.2	131.0 ± 40.9	124.3 ± 38.3
Total cholesterol (mg/dL)	200.8 ± 42.9	202.3 ± 43.1	210.2 ± 46.6	206.2 ± 46.9
HDL-C (mg/dL)	52.4 ± 15.3	50.2 ± 15.7	47.6 ± 15.5	50.6 ± 15.6
Triglycerides (mg/dL)	126 (74–151)	143 (83–171)	149 (84–178)	137 (79–163)

*Data are presented as frequency and percentage n (%), mean ± standard deviation, or mean (range). BMI: body mass index. BP: blood pressure. HPL: hyperlipidemia. HTG: hypertriglyceridemia. LDL-C: low density lipoprotein cholesterol. HDL-C: high density lipoprotein cholesterol.

For the non-cigarette tobacco products, the prevalence of current use of cigar, pipe, and smokeless tobacco, in the overall population is 2.1% (991), 1.2% (523), and 2.2% (1375), respectively. Data on e-cigarette use is available for FHS 3^rd^ generation, MESA, CARDIA, REGARDS, and HCHS/SOL with 191, 31, 219, 331, and 932 users (current and former), respectively. Table 5 shows the prevalence of non-cigarette tobacco product use status stratified by cigarette smoking status. The prevalence of cigars, pipes, and smokeless tobacco use is 2.5%, 1.2%, and 2.0%, respectively, among participants who currently smoke combustible cigarettes. The prevalence among participants who formerly smoked cigarettes is 4.5% for cigar, 4.5% for pipe, and 7.3% for smokeless tobacco use. Among individuals who had never smoked cigarettes, the prevalence of each of the non-cigarette tobacco products is <2%.

**Table 5:** Non-traditional tobacco use status across combustible cigarette smoking status in the Cross-Cohort Collaboration-Tobacco dataset

*Non-traditional tobacco use*	*Combustible cigarette smoking status*			
	*Never smoker n (%)*	*Former smoker n (%)*	*Current smoker n (%)*	*Total n (%)*
**Cigar**				
Never	22175 (96.2)	10797 (85.8)	10665 (92.6)	43637 (92.7)
Former	469 (2.0)	1455 (11.6)	521 (4.5)	2445 (5.2)
Current	389 (1.7)	319 (2.5)	283 (2.5)	991 (2.1)
**Pipe**				
Never	20607 (97.2)	9341 (86.6)	10170 (93.3)	40118 (93.6)
Former	398 (1.9)	1238 (11.5)	596 (4.5)	2232 (5.2)
Current	190 (0.9)	202 (1.9)	131 (1.2)	523 (1.2)
**Smokeless**				
Never	28526 (94.8)	18560 (86.9)	10992 (90.7)	58078 (91.4)
Former	978 (3.2)	2217 (10.3)	881 (7.3)	4076 (6.4)
Current	557 (1.8)	576 (2.7)	242 (2.0)	1375 (2.2)

Inflammatory markers, a priority area for CCC-Tobacco, were evaluated at baseline and during follow-up. The number of measurements of each inflammatory marker is given in Supplementary file Table 3.

## DISCUSSION

The CCC is a research initiative that involves pooling data from several existing prospective cohort studies in the US and Brazil to create a large and diverse dataset capable of leveraging the power in addressing questions that would be unanswerable or otherwise underpowered using a single cohort. The CCC’s core focus is on harmonizing data collected from the various studies to ensure consistency and reliability of the findings. The CCC-Tobacco dataset will enable the examination of the association of traditional and non-traditional tobacco product use with subclinical and clinical CVD in adults, with a particular focus on understudied minority groups. Moreover, because of the large sample size, the cohort will make possible for the first time to study the differential impact of smoking as well as the health effects of non-traditional tobacco products in different population subgroups.

The CCC-Tobacco is significant for several reasons. Despite the rise in usage of non-traditional tobacco products such as cigars, pipes, e-cigarettes, and smokeless tobacco, well-powered studies on their long-term impact on cardiovascular health in a wellcharacterized population are limited. Furthermore, to the best of our knowledge, no prior study has systematically explored the relationship between cigars, pipes, and smokeless tobacco and multiple domains of subclinical markers of CVD, as well as the extent to which cardiovascular outcomes are caused by these non-cigarette tobacco products and mediated by these subclinical markers, and how they may vary among different subgroups. The CCC-Tobacco data will enable us to identify new biomarkers of cardiovascular harm associated with combustible cigarette use and the extent to which these biomarkers mediate cardiovascular risk. Additionally, using the CCC-Tobacco dataset, which has extensive data on non-cigarette tobacco products, we will be able to link the use of non-cigarette tobacco products to already established markers of cardiovascular harm including markers of subclinical inflammation (high sensitivity C-reactive protein and interleukin-6) and novel markers such as CAC^24-29^.

The FDA considers the study of the health effects of alternative tobacco products using longitudinal data as a top research priority^30^. Our work will help elucidate the health effects of these non-cigarette tobacco products with respect to the hypothesized risk continuum^31-33^. Therefore, our work with the CCC-Tobacco could prove vital to the regulatory authority of the FDA and other policy initiatives and recommendation regarding non-cigarette tobacco products in a way that is deemed appropriate for the protection of public health. The importance of addressing CVD as a major contributor to morbidity and mortality is paramount to improving public health. The approach and descriptive findings presented here demonstrate the unique strength of the CCC-Tobacco to provide crucial information that can inform public health strategies and policies regarding non-cigarette tobacco product regulation.

### Challenges and limitations

While this article seeks to provide insight into the logistical process of data acquisition and harmonization in addition to an insight into the characteristics of the pooled dataset, we also discuss challenges in our work. Challenges encountered during the early phases include those associated with establishing contact with study personnel and keeping study collaborators engaged. Additionally, the lack of an existing streamlined process for data transfer and completed mandatory data-use contracts led to a largely unpredictable workflow resulting in delays. On a few occasions, following the approval process, datasets were delivered to the processing site in inaccessible formats. Additionally, the CCC-Tobacco database has some limitations. First, the observational study design leads to the potential for residual confounding and limitations in the ability to establish causal relations. Secondly, age distributions limit the ability to generalize to children and young adults smoking patterns and associations. Third, despite the large sample size, the number of individuals using non-traditional tobacco products was still quite modest. Lastly, the studies did not routinely collect data on individuals who were sexual or gender minorities or who used a variety of illicit drugs.

### Future perspectives

We envision that the experience and challenges reported in establishing the CCC-Tobacco will serve as a learning opportunity for other cross-cohort work and provide a potential framework for additional future cross collaboration and data sharing between NHLBI studies. Our dataset will potentially serve as an epidemiological resource for the tobacco research community at large. Our dataset will serve as a rich epidemiological resource for other working groups in the CCC and the research community at large. Our approach also provides considerable room for expansion of the current dataset. CCC-Tobacco can be easily expanded to include other risk factors and cohorts, including advanced biomarkers and Omics measures, and results can be compared with other consortia like the Emerging Risk Factors Collaboration^34,35^.

Furthermore, we plan to continue to harmonize all tobacco use at each additional visit beyond the baseline study visit of each cohort in order to provide unprecedented longitudinal tobacco use data to expand our analysis into the study of tobacco use transitions (product switching and changes in use intensity) and their relative association with subclinical and clinical CVD. Lastly, cohorts (MESA, FHS 3^rd^ generation, CARDIA, REGARDS, and HCHS-SOL) starting to collect data on new tobacco products such as e-cigarette at follow-up, will expand our knowledge regarding the health effects of these products.

## CONCLUSIONS

The CCC-Tobacco dataset, with its large sample size, long-term follow up, diverse study population, and encompassing multiple subclinical features and clinical CVD events, aims to expand our knowledge regarding traditional and non-traditional tobacco products and their association with subclinical and clinical CVD. We aim to identify novel biomarkers of cardiovascular harm associated with combustible cigarette and non-cigarette tobacco product use^36^. The large sample size of women and other underrepresented groups allows for research in these historically understudied groups. Future iterations of this project, by providing data on long-term tobacco use and tobacco produce use transitions, could provide important information on how changes in tobacco use patterns influence markers of subclinical cardiovascular injury and CVD risk. The findings from the CCC-Tobacco will therefore provide the FDA with new and pertinent knowledge that would inform regulation of non-cigarette tobacco products. Ultimately, our aim is to obtain new information regarding the cardiovascular impact of non-traditional tobacco products and to deliver actionable results to the tobacco regulatory science community.

## Data Availability

The data supporting this research are available from the corresponding author on reasonable request.

## References

[1] World Health Organization Cardiovascular diseases.

[2] Tsao CW, Aday AW, Almarzooq ZI (2023). Heart disease and stroke statistics-2023 update: a report from the American Heart Association. Circulation.

[3] Aune D, Schlesinger S, Norat T, Riboli E (2018). Tobacco smoking and the risk of sudden cardiac death: a systematic review and meta-analysis of prospective studies. Eur J Epidemiol.

[4] Roy A, Rawal I, Jabbour S, Prabhakaran D, Prabhakaran D, Anand S, Gaziano TA, Mbanya JC, Wu Y, Nugent R (2017). Tobacco and Cardiovascular Disease: A Summary of Evidence. Cardiovascular, Respiratory, and Related Disorders.

[5] Dai X, Gakidou E, Lopez AD (2022). Evolution of the global smoking epidemic over the past half century: strengthening the evidence base for policy action. Tob Control.

[6] Cornelius ME, Loretan CG, Wang TW, Jamal A, Homa DM (2022). Tobacco product use among adults - United States, 2020. MMWR Morb Mortal Wkly Rep.

[7] National Center for Chronic Disease Prevention and Health Promotion (US) Office on Smoking and Health (2014). The Health Consequences of Smoking—50 Years of Progress: A Report of the Surgeon General.

[8] Wang TW, Kenemer B, Tynan MA, Singh T, King B (2016). Consumption of combustible and smokeless tobacco - United States, 2000-2015. MMWR Morb Mortal Wkly Rep.

[9] Kasza KA, Ambrose BK, Conway KP (2017). Tobacco-product use by adults and youths in the United States in 2013 and 2014. N Engl J Med.

[10] Obisesan OH, Osei AD, Uddin SMI (2020). Trends in e-cigarette use in adults in the United States, 2016-2018. JAMA Intern Med.

[11] Park-Lee E, Ren C, Cooper M, Cornelius M, Jamal A, Cullen KA (2022). Tobacco product use among middle and high school students - United States, 2022. MMWR Morb Mortal Wkly Rep.

[12] Rahmanian SD, Diaz PT, Wewers ME (2011). Tobacco use and cessation among women: research and treatment-related issues. J Womens Health (Larchmt).

[13] Cullen J, Mowery P, Delnevo C (2011). Seven-year patterns in US cigar use epidemiology among young adults aged 18-25 years: a focus on race/ethnicity and brand. Am J Public Health.

[14] Boakye E, Osuji N, Erhabor J (2022). Assessment of patterns in e-cigarette use among adults in the US, 2017-2020. JAMA Netw Open.

[15] Xie W, Kathuria H, Galiatsatos P (2020). Association of electronic cigarette use with incident respiratory conditions among US adults from 2013 to 2018. JAMA Netw Open.

[16] Berlowitz JB, Xie W, Harlow AF (2023). Cigarettee-cigarette transitions and respiratory symptom development. Am J Prev Med.

[17] Berlowitz JB, Xie W, Harlow AF (2022). E-cigarette use and risk of cardiovascular disease: a longitudinal analysis of the PATH Study (2013-2019). Circulation.

[18] Collaborative Health Studies Coordinating Center Cross-Cohort Collaboration Consortium.

[19] Food and Drug Administration, HHS (2016). Deeming Tobacco Products To Be Subject to the Federal Food, Drug, and Cosmetic Act, as Amended by the Family Smoking Prevention and Tobacco Control Act; Restrictions on the Sale and Distribution of Tobacco Products and Required Warning Statements for Tobacco Products. Final rule. Fed Regist.

[20] Fortier I, Raina P, Van den Heuvel ER (2017). Maelstrom Research guidelines for rigorous retrospective data harmonization. Int J Epidemiol.

[21] Stilp AM, Emery LS, Broome JG (2021). A System for Phenotype Harmonization in the National Heart, Lung, and Blood Institute Trans-Omics for Precision Medicine (TOPMed) Program. Am J Epidemiol.

[22] Wilkins JT, Karmali KN, Huffman MD (2015). Data Resource Profile: The Cardiovascular Disease Lifetime Risk Pooling Project. Int J Epidemiol.

[23] Mishra GD, Chung HF, Pandeya N (2016). The InterLACE study: design, data harmonization and characteristics across 20 studies on women’s health. Maturitas.

[24] McEvoy JW, Nasir K, DeFilippis AP (2015). Relationship of cigarette smoking with inflammation and subclinical vascular disease: the Multi-Ethnic Study of Atherosclerosis. Arterioscler Thromb Vasc Biol.

[25] Al Rifai M, DeFilippis AP, McEvoy JW (2017). The relationship between smoking intensity and subclinical cardiovascular injury: The Multi-Ethnic Study of Atherosclerosis (MESA). Atherosclerosis.

[26] Kianoush S, Yakoob MY, Al-Rifai M (2017). Associations of cigarette smoking with subclinical inflammation and atherosclerosis: ELSA-Brasil (The Brazilian Longitudinal Study of Adult Health). J Am Heart Assoc.

[27] Kianoush S, Bittencourt MS, Lotufo PA (2017). Association between smoking and Serum GlycA and High-Sensitivity C-Reactive Protein Levels: The Multi-Ethnic Study of Atherosclerosis (MESA) and Brazilian Longitudinal Study of Adult Health (ELSA-Brasil). J Am Heart Assoc.

[28] Tibuakuu M, Kamimura D, Kianoush S (2017). The association between cigarette smoking and inflammation: the Genetic Epidemiology Network of Arteriopathy (GENOA) study. PLoS One.

[29] Jones MR, Magid HS, Al-Rifai M (2016). Secondhand smoke exposure and subclinical cardiovascular disease: the Multi-Ethnic Study of Atherosclerosis. J Am Heart Assoc.

[30] National Institutes of Health, U.S. Dept of Health and Human Services Research Priorities.

[31] El-Toukhy S, Choi K (2016). A risk-continuum categorization of product use among US youth tobacco users. Nicotine Tob Res.

[32] Zeller M, Hatsukami D, Strategic Dialogue on Tobacco Harm Reduction Group (2009). The Strategic Dialogue on Tobacco Harm Reduction: a vision and blueprint for action in the US. Tob Control.

[33] Solyst J (2012). Toward a comprehensive policy on nicotine delivery products and harm reduction. Food Drug Law J.

[34] Danesh J, Erqou S, Walker M, Emerging Risk Factors Collaboration (2007). The Emerging Risk Factors Collaboration: analysis of individual data on lipid, inflammatory and other markers in over 1.1 million participants in 104 prospective studies of cardiovascular diseases. Eur J Epidemiol.

[35] University of Cambridge Cardiovascular Epidemiology Unit: ERFC.

[36] Conklin DJ, Schick S, Blaha MJ (2019). Cardiovascular injury induced by tobacco products: assessment of risk factors and biomarkers of harm. A Tobacco Centers of Regulatory Science compilation. Am J Physiol Heart Circ Physiol.

