# Supplementary material for: Investigating the association of traditional and non-traditional tobacco product use with subclinical and clinical cardiovascular disease: The Cross-Cohort Collaboration-Tobacco working group rationale, design, and methodology
Source: Tob Induc Dis. 2023 Jul 7;21:89. doi: 10.18332/tid/166517 (PMC10326890; doi:10.18332/tid/166517)
Supplement: Supplementary file 1 [file TID-21-89-s1.pdf]

**Supplementary table 1. Weblink for the Twenty-three Participating Cohorts of the Cross-Cohort Collaboration-Tobacco Dataset**	
**Participating cohort**	Website link
**Atherosclerosis Risk in Communities Study (ARIC)**	https://sites.cscc.unc.edu/aric/desc
**Coronary Artery Risk Development in Young Adults (CARDIA)**	https://www.cardia.dopm.uab.edu/
**Cardiovascular Health Study (CHS)**	https://chs-nhlbi.org/
**Dallas Heart Study (DHS)**	https://www.utsouthwestern.edu/education/medical-school/departments/internal-medicine/research/dallas-heart/
**Framingham Heart Study (FHS)**	https://www.framinghamheartstudy.org/
**Hispanic Community Health Study/Study of Latinos (HCHS-SOL)**	https://sites.cscc.unc.edu/hchs/
**Jackson Heart Study (JHS)**	https://www.jacksonheartstudy.org/
**Multi-Ethnic Study of Atherosclerosis (MESA)**	https://www.mesa-nhlbi.org/
**The Multiple Risk Factor Intervention Trial (MRFIT)**	https://biolincc.nhlbi.nih.gov/studies/mrfit/
**Reasons for Geographic and Racial Differences in Stroke (REGARDS)**	https://www.uab.edu/soph/regardsstudy/
**Strong Heart Study (SHS)**	https://strongheartstudy.org/
**Baltimore Longitudinal Study of Aging (BLSA)**	https://www.blsa.nih.gov/
**Chronic Renal Insufficiency Cohort Study (CRIC)**	http://www.cristudy.org/Chronic-Kidney-Disease/Chronic-Renal-Insufficiency-Cohort-Study/
**Brazilian Longitudinal Study of Adult Health (ELSA-Brasil)**	https://pubmed.ncbi.nlm.nih.gov/22234482/
**Genetics of Lipid Lowering Drugs and Diet Network (GOLDN)**	https://www.ncbi.nlm.nih.gov/projects/gap/cgi-bin/study.cgi?study_id=phs000741.v1.p1
**Health, Aging and Body Composition Study (Health ABC)**	https://healthabc.nia.nih.gov/
**The Osteoporotic Fractures in Men Study (MrOS)**	https://mrosonline.ucsf.edu/
**Rancho Bernardo Study (RBS) of Healthy Aging**	https://knit.ucsd.edu/ranchobernardostudy/
**The Study of Osteoporotic Fractures (SOF)**	https://sofonline.ucsf.edu/
**Study of Women's Health Across the Nation (SWAN)**	https://www.swanstudy.org
**Women's Health Initiative (WHI)**	https://www.whi.org/

Supplementary table 2. Distribution of Race and Ethnicity Across Combustible Cigarette Smoking Status in the Cross-Cohort Collaboration-Tobacco Dataset

	**Smoking status**			
	**Never**	**Former**	**Current**	**Total**
**Race and ethnicity, N (%)**				
**White**	105,907 (47.08)	89,552 (39.81)	29,498 (13.11)	224,957 (100.00)
**African - American**	24,576 (51.18)	14,458 (30.11)	8,981 (18.70)	48,015 (100.00)
**Asian**	4,080 (72.92)	1,274 (22.77)	241 (4.31)	5,595 (100.00)
**Hispanic**	11,743 (59.68)	4,348 (22.10)	3,586 (18.22)	19,677 (100.00)
**American Indian or Alaskan**	3,824 (44.13)	3,026 (34.92)	1,815 (20.95)	8,665 (100.00)
**other**	105 (24.25)	138 (31.87)	190 (4.31)	433 (100.00)

Supplementary table 3. Inflammatory Markers Measurements at Baseline and Follow-up in the Cross-Cohort Collaboration-Tobacco Dataset

Inflammatory markers	Baseline (N)	Follow up (N)
**Interleukin-6**	33,258	35,583
**HsCRP**	137,098	104,924
**D-dimer**	12,304	8,938
**Fibrinogen**	68,481	38,251
**CAC score measurements**	22,936	15,874
**Carotid plaque**	3,745	15,539
**CIMT**	8,716	12,429
**Ankle-brachial index**	43,123	20,021
**GlycA**	7,108	1,142
**Factor vii**	10,036	1,508

HsCRP: high-sensitive C-reactive protein; CAC: coronary artery calcium; CIMT: coronary intima-media thickness; ABI: ankle-brachial index.

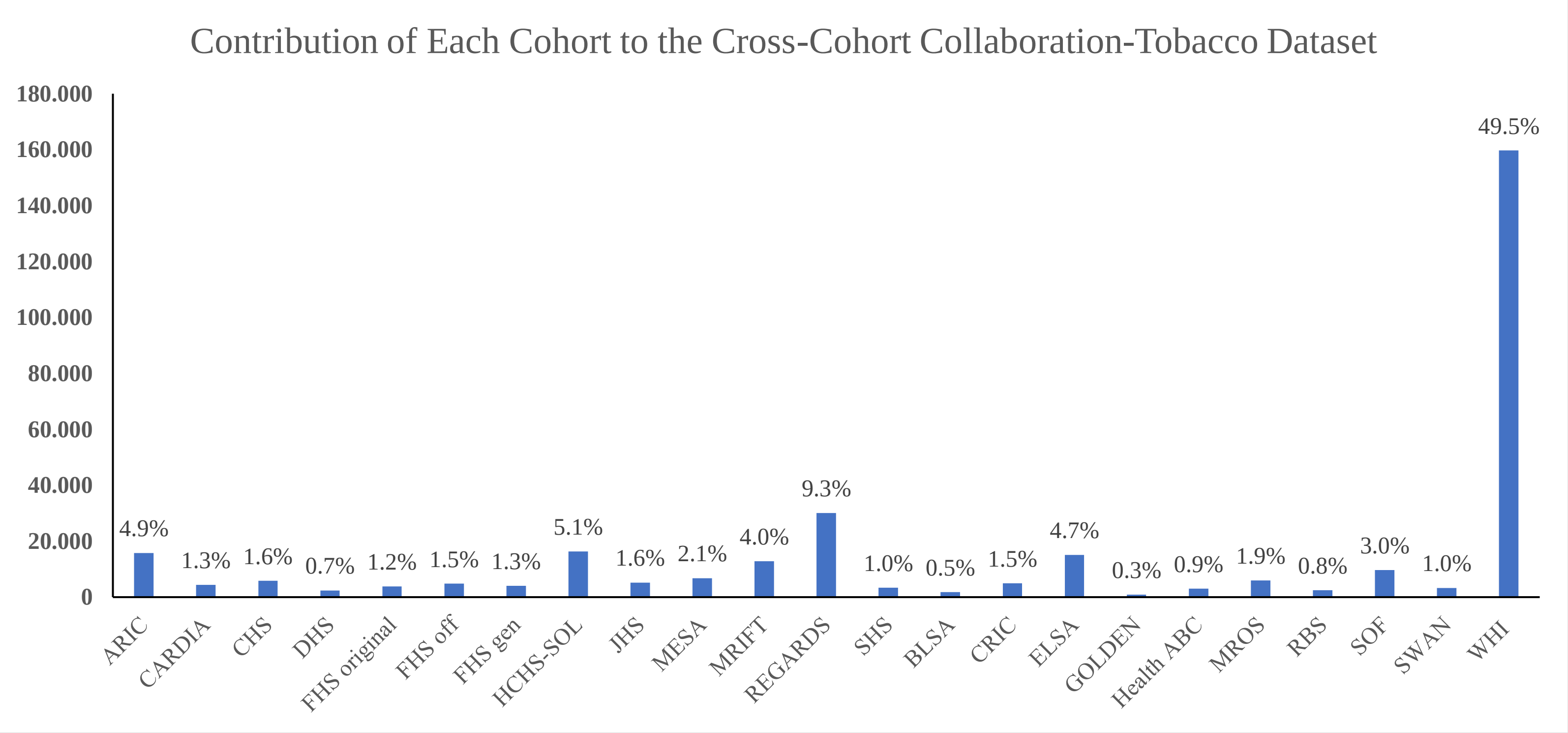

Supplementary Figure 1. Contribution of Each Cohort to the Cross-Cohort Collaboration-Tobacco Dataset. ARIC: Atherosclerosis Risk in Communities Study; CARDIA: Coronary Artery Risk Development in Young Adults Study; CHS: Cardiovascular Health Study; DHS: Dallas Heart Study; FHS: Framingham Heart Study; HCHS/SOL: Hispanic Community Health Study/Study of Latinos; JHS Jackson Heart Study; MESA: Multi-Ethnic Study of Atherosclerosis; MRFIT: Multiple Risk Factor Intervention Trial; REGARDS: the Reasons for Geographic and Racial Differences in Stroke Study; SHS: the Strong Heart Study; BMI: body mass

index; BP: blood pressure; HPL: hyperlipidemia; HTG: hypertriglyceridemia; LDL-C: low density lipoprotein cholesterol; HDL-C: high density lipoprotein cholesterol

© 2023 Tasdighi E. et al.
